# Caring Climate and Support, Mental Health, and Academic Adjustment: Effects from a Cluster Randomized Controlled Trial in Upper Secondary Schools in Norway

**DOI:** 10.3390/ijerph20227033

**Published:** 2023-11-08

**Authors:** Torill Bogsnes Larsen, Helga Bjørnøy Urke, Sara Madeleine Kristensen, Frida Kathrine Sofie Mathisen

**Affiliations:** Department of Health Promotion and Development, University of Bergen, 5009 Bergen, Norway; helga.urke@uib.no (H.B.U.); madeleine.kristensen@uib.no (S.M.K.); frida.mathisen@uib.no (F.K.S.M.)

**Keywords:** cluster randomized controlled trial, multi-tier intervention, dream school program, mental health support team, psychosocial environment

## Abstract

This cluster randomized controlled trial (RCT) examined the effect of a three year follow up of a multi-tier intervention aiming at improving the psychosocial environment in upper secondary schools in Norway. Two intervention conditions were tested: a universal single-tier intervention focused on improving the psychosocial school climate, the dream school program (DSP), and a multi-tier intervention combining the DSP with a targeted measure, the mental health support team (MHST). A total of 2203 students responded to the baseline survey. Of the 2203 pupils, 1884 responded to the first follow-up survey (year 1), 1287 pupils to the second (year 2), and 756 pupils to the third (year 3). The direct and indirect effects on school completion were analyzed using a multi-level linear mixed model. The results showed no significant effects of either the DSP or the DSP and MHST in combination on support, the school climate, mental health, or academic adjustment. We found no significant effect of the interventions on the proportion who had completed school or were in training (the three groups varied between 76.6% and 77.8%). Future similar studies should be attentive to the potential challenges of implementing RCTs in the school setting. Furthermore, the long-term effects of school interventions on the constructs included in this study could be difficult to capture due to the complexity of the phenomena. The implications of these findings are discussed.

## 1. Introduction

The labor market of today places ever greater demands on formal competence, and successful completion of upper secondary education is an important basis for further education, health, and integration into work life. Completing upper secondary schooling has been related to gaining higher education [1,2], reduced risk of later economic hardship [3], better mental and physical health, and greater life satisfaction [4,5]. In contrast, young people who do not complete school, or who are left out of both education and work for a longer period, risk permanent exclusion from work life, more mental health problems, as well as reduced health over the life course.

Studies have found that students who are not socially integrated, for instance, those who do not experience school belonging or a caring school climate, or are lonely, are more likely to have lower academic functioning [6], feel reduced life satisfaction, experience difficulties in relation to their mental health [7], achieve poorer academic outcomes [8], and have an increased risk of dropping out of school [9,10,11]. In their review of upper secondary school dropout, Lillejord et al. [12] highlighted supportive teacher and peer relationships in the learning environment and follow up of students at risk as a key prevention factor, and further suggested that dropout prevention efforts should be characterized via care work. Thus, one step towards increasing upper secondary school thriving and completion is ensuring inclusive psychosocial learning environments where adolescents want to be, learn, and develop.

The present study reported results from the COMPLETE project, a randomized controlled trial (RCT) that implemented a universal intervention, the dream school program (DSP), and a selective/indicated intervention, mental health support teams (MHSTs), in upper secondary schools in Norway with the aim of increasing psychosocial thriving and academic adjustment through systematic work with the psychosocial learning environment [13].

### 1.1. Theoretical Background

In the broadest sense, the psychosocial learning environment concerns the interpersonal relationships at school and how these affect the students’ experience of the learning situation [14]. The significance of the psychosocial dimension of the school environment is founded in the theoretical proposition that having close relationships fosters a sense of belonging, which the theory of belonging and self-determination postulates to be a basic human need [15,16]. Belonging assists the building of positive relationships between people and institutions, whereby both parties contribute to the relationship [17]. When the need to belong has been satisfied, it can result in better socioemotional health and wellbeing [15]. Therefore, an inclusive school environment can engender belonging through caring and supportive relationships with teachers and peers.

In addition to relatedness (i.e., the need to belong), the self-determination theory argues that satisfying the psychological needs for autonomy and competence are particularly important for motivation and performance in school [15,18]. Autonomy and competence satisfaction may therefore be crucial in upper secondary school, as poor academic performance is a strong contributor to dropout [2,6,19]. The need for autonomy refers to the experience of being able to influence and make one’s own independent choices [15]. For example, people in an autonomy-supportive psychosocial learning environment provide students meaningful rationales for their learning, recognize, and address their negative feelings, communicate in non-controlling ways, provide relevant choices, and cultivate the students’ motivational resources [20]. Having an autonomy-supportive teacher increases the students’ autonomous motivation and wellbeing [21] and reduces their anxiety or depression [22]. The need for competence is associated with a higher level of academic self-concept and achievement motivation [23]. As this need is satisfied when students feel a certain control of their academic performance, a structured learning environment is assumed to promote competence [24].

According to the stage environment fit theory [25], the transition from secondary school to upper secondary education is perceived as a risk factor for adolescent school- and general wellbeing through factors, such as frustration, brought about by the psychological needs for belonging, autonomy, and competence [26]. For example, this transition can cause young people to lose important social relationships [27], and for some establishing new relationships can be a demanding task. Furthermore, previous research has shown that students often experience a loss of autonomy when transitioning to upper secondary school, which results in reduced commitment and motivation [25,28]. Related to this, the transition from lower to upper secondary school also entails greater and different academic demands that, at least, can temporarily compromise feelings of competence in adolescents, for example through poorer academic achievement [25]. Facilitating a positive psychosocial learning environment at the start of the school year to counteract loneliness, loss of motivation, and decreased wellbeing can ease the transition from lower to upper secondary school [29].

### 1.2. Previous Research on School-Based Interventions to Improve the Psychosocial Learning Environment and Promote Completion

Schools can be seen as key environments for the promotion of adolescents’ mental health, academic performance, and belonging, and school-based programs are able to reach many youths from different family backgrounds. However, Poling et al. [30] found, in their review on teacher–student support, that few studies had implemented interventions providing students support directly beyond the universal level, and that few studies had been conducted within secondary schools and beyond. In the COMPLETE project, an important aim was to combine the universal and targeted approaches in the upper secondary school setting. The DSP is focused on creating a positive psychosocial environment, which we know impacts learning [31,32,33], and the MHST is aimed at improving individual experience, as well as students’ ability to cope with school requirements and ultimately complete upper secondary education.

In a previous study from the COMPLETE project, no significant effects were observed of neither the DSP nor the DSP and MHST in combination on loneliness in the first follow-up (8 months after baseline), but a small effect was identified on mental health problems for girls [23]. Similar to the aims of the DPS and MHST, Allen et al. [34] found, in their systematic review on school belonging, that fostering school belonging through interventions building on students’ strengths and promoting positive interaction between students and between students and staff had several positive outcomes, like reduced bullying and depression levels. In addition, this review found that students at risk or vulnerable students in need of mental health support also benefitted from interventions addressing school belonging. This is also supported by other research, showing that supportive social relationships in school and feelings of school belonging are positively associated with several outcomes, like social, psychological, and academic functioning [35,36,37], self-esteem [38], positive academic outcomes [8], subjective wellbeing, and life satisfaction [7,31]. The importance of positive relations within the school environment was also found in a systematic review published by Krane et al. [9], showing that teacher–student relationships characterized by support, care, respect, and trust could promote good psychological functioning and prevent student psychological distress and dropout.

Thus, focusing on building good relationships and environments, through the involvement and extensive use of young people’s own resources, can strengthen their sense of meaning, motivation, and belonging to their schools, which increases the likelihood of completion and prevents dropouts.

### 1.3. The Dream School Program (DSP)

The DSP is a universal model aimed at improving the psychosocial environment through a whole-of-school approach developed by the non-governmental organization Adults for Children. With its universal approach, it represents the first tier of prevention work [39]. The goals of the DSP, as formulated by Adults for Children [40], are to: (a) establish frameworks and tools for holistic work with the psychosocial learning environment in the school, (b) increase the competence of employees to create a good psychosocial environment, (c) strengthen the relationship between students and between students and staff, (d) strengthen students’ sense of belonging, participation, self-efficacy, and motivation, (e) increase students’ motivation to complete and pass school, and (f) use student mentors as resources in systematic work to promote a good psychosocial environment.

The DSP was administered to 2127 students (in 143 classes) across 12 schools. The number of year 1 classes in each school varied from two to six in the smallest schools and from eighteen to thirty in the largest. The core elements of the program are dream classes 1 and 2, which are three-hour sessions focusing on the class climate for pupils in year 1 and are carried out in the first or second week after the start of school and at the beginning of the spring semester. Student mentors are involved in the implementation of these meetings. These are older students, who after training by Adults for Children monitor the psychosocial environment at school. There were 313 student mentors at the 12 schools that introduced the DSP in the autumn of 2016. The contact teachers were also trained by Adults for Children and involved in the implementation of the dream class. They were responsible for implementing the action plan that the class creates and important partners for the student mentors in their work with the class. At each school, a resource group was set up, with school managers, teachers, students, and other employees responsible for conducting the DSP in their school [13,41].

### 1.4. The Mental Health Support Team (MHST)

A MHST is an indicative and selective measure to prevent dropouts. It represents tiers 2 and 3 of prevention, whereby a small group of students receives interventions (tier 2), and intensive intervention efforts are made for at-risk students (tier 3) [39]. A MHST is intended for the psychosocial follow-up of young people at risk of dropping out of upper secondary education. The purpose of this measure is to ensure that such young people receive close follow-up by maintaining a continuous presence at school. Furthermore, systematic transition work targets the phases between lower and upper secondary school and between the individual grade levels of upper secondary school.

A MHST is organized as a multidisciplinary and co-located team to strengthen the prevention work of student services. These teams have had a slightly different composition, but the school health nurse, school follow-up adviser, and social counselor have central functions within these teams. In addition, pedagogical psychological services, labor market counselors in school, and the social educator have been a part of teams at some schools. Student services have been provided with resources to cover a 50% position at each project school to act as coordinator for the team. The intention of this project was for the teams to sit together and have an open-door policy to students, as well as work on school attendance, facilitate transition, and for a nurse to conduct individual student assessments using the Kidscreen tool [42].

### 1.5. The Present Study

The present study is a follow-up study partly building on the first effect study of the COMPLETE project that examined effects on loneliness and mental health problems in the first year of upper secondary school [23]. The COMPLETE project followed students over three years of upper secondary school, and in the present study the aim was to examine whether the DSP and MHST intervention measures impacted the students’ perceptions of their psychosocial learning environment, mental health, and academic adjustment across and after three years of upper secondary school. Specifically, we investigated whether the DSP alone and the DSP and MHST in combination influenced the students’ 1) perceived psychosocial environment measured in terms of classmate support (i.e., class belonging), the school climate, and teacher support; 2) mental health measured as psychological distress (i.e., symptoms of anxiety and depression), life satisfaction, and loneliness; and 3) academic adjustment measured as grade point average, absenteeism, and school completion. The DSP and MHST were designed to raise the level of support and care in the students’ psychosocial learning environment and target at-risk students with intensive follow-ups and support. Thus, these measures were assumed to increase the overall quality of the psychosocial learning environment, resulting in improved mental health and academic adjustment in upper secondary school.

## 2. Material and Methods

### 2.1. Study Design

COMPLETE is a school-based, three-armed longitudinal cluster RCT in sixteen upper secondary schools located in four counties of Norway. Through the measures of DSP and MHST, the aim was to facilitate systematic work on the psychosocial learning environment and follow-up of pupils. We the evaluated the effectiveness of the DSP and MHST interventions from year one (Y1) to year three (Y3) on students’ perception of the psychosocial learning environment, mental health, and academic adjustment. Teacher support, classmate support, a caring school climate, psychological distress, and life satisfaction were measured using commonly accepted scales and questionnaires. There were three study groups: two intervention groups and a control group. The trial started in August 2016 and ended in June 2019. It followed the students from their commencement of upper secondary school to graduation. This study was unblinded (see Larsen et al. [13] for more details).

### 2.2. Sample

All Norwegian-speaking students starting the first year of upper secondary school in the 16 included schools were invited to participate. Those under the age of 16 years needed their parental or guardian consent to participate. Among the 820 students who needed consent, 72% provided feedback, and 70% gave content for the student to participate. A total of 2203 students from either a general or a vocational study track responded to the baseline survey (at the start of the first school year). Of the 2203 pupils, 1884 responded to the first follow-up survey (in the spring of year 1), 1287 pupils to the second (spring of year 2), and 756 pupils to the third (spring of year 3). The number of survey respondents decreased over time from the start to the end of year 3. Among other reasons, this was because students who attend a vocational education program are most often in apprenticeships after two years of upper secondary school. The students from vocational subjects, who were not present at the schools in the third year, were contacted via SMS and prompted to complete the survey online. However, very few responded. We also found that at the final measurement point, fewer students responded to the survey than were registered in each class. Moreover, at this time, fewer students who had not been present at the data collection time subsequently responded.

### 2.3. Randomization

To ensure an equal balance of intervention and control groups in each county, stratified randomization via county was practiced. All schools were given a random number ranging from 0 to 1 using a randomization command in Stata 14 statistical software. Stata generated a random number based on a chosen key. Next, the schools were sorted according to the magnitude of the random number in each stratum. The school with the highest number was allocated to intervention group 1, I1 (DSP only), the one with the second highest number to intervention group 2, I2 (DSP and MHST), and the third highest to the control group, C. This procedure was repeated until all schools in each stratum were appointed to a group.

### 2.4. Measures

#### 2.4.1. Teacher Support

Teacher support was assessed using a short version of the Learning Climate Questionnaire (LCQ) [43]. The LCQ consists of five statements about the teachers with the response options “Strongly disagree,”, “Disagree,”, “Neither agree nor disagree,”, “Agree,”, and “Strongly agree.”. Among other items, the students were asked about their experience of the teachers, e.g., “The teachers give me options.”. The students’ answers were converted into one variable, so that a higher number indicates greater teacher support. The possible average ranged between the values of 1 and 5 [43].

#### 2.4.2. Classmate Support

Classmate support was assessed with the teacher and classmate support scale [43] and measured using five statements about the class and attending school, with the response options “Strongly disagree,”, “Disagree,”, “Neither agree nor disagree,”, “Agree,”, and “Strongly agree.”. Among other topics, the students were asked about their interpersonal experiences in the classroom, e.g., “The students in my class like being with each other.”. The students’ answers to the five statements were converted into one variable. The possible average ranged from 1 to 5, wherein a higher number was interpreted as greater classmate support [43].

#### 2.4.3. Caring Climate

Caring school climate was mapped using the caring climate scale [44], including 13 statements about the relationship with the teachers, for which the answer options were “Strongly disagree,”, “Disagree,”, “Don’t know,”, “Agree,”, and “Strongly agree.”. An example item is “Teachers are kind to students.”. As for both teacher support and class affiliation, the students’ answers to the questions were converted into one variable, so that a higher number was interpreted as students perceiving a more caring climate. The possible average ranged from 1 to 5 [44].

#### 2.4.4. Psychological Distress

Psychological distress was measured using the short form of the Symptom Checklist (SCL-5) [45], including five indicators of symptoms of anxiety and depression (e.g., “Constantly afraid and anxious” and “Feeling down and sad”). The students were asked to report the degree to which they had been troubled by any of these in the previous 14 days. The answer categories given were “Not bothered,”, “Somewhat bothered,”, “Quite bothered,”, and “Very bothered.”. The possible score for psychological distress thus ranged from 1 to 4, and a higher score implied greater psychological distress [45].

#### 2.4.5. Life Satisfaction

Life satisfaction was measured with the Multi-dimensional Student’s Life Satisfaction Scale by Huebner [46], including nine statements about life (e.g., “My life is going well”). Students were asked to report how often they would have agreed in recent weeks. The response categories were “Never,”, “Occasionally,”, “Often,”, and “Almost always.”. The possible score for life satisfaction thus ranged from 1 to 4, and an increased score indicated an improvement in life satisfaction [46,47].

#### 2.4.6. Loneliness

Loneliness was measured using the Loneliness scale (adapted for a Norwegian context by Mittelmark et al. [48]), including six statements about how students felt (e.g., “I often feel lonely”). The answer categories were “Not at all,”, “To a small extent,”, “To some extent,”, “To a fairly large extent,”, and “To a very large extent.”. The possible score for loneliness thus ranged from 1 to 5, and an increase in the score implied more loneliness [48].

#### 2.4.7. Grade Point Average

The students’ grades were obtained from registry data in the counties and included academic results from all the subjects the students participated in during the school year. The grade point average in this study was based on the average grade in these subjects and ranged from 1 (fail) to 6 (the highest grade).

#### 2.4.8. Absenteeism

Student absenteeism data were obtained from county school registries and included the total hours and days absent from school for each school year per student.

#### 2.4.9. Completion

Completion data were based on county registry data for the participating schools. All pupils were surveyed regarding their progress towards completion after three years, whether they had changed schools or program areas during the period, and those who were registered as having left were also categorized according to whether they were active or not. For students from vocational subjects, it was also recorded whether they had been offered an apprenticeship from August 2019.

#### 2.4.10. Sex

The participants’ sex was collected from registry data, wherein boys were coded as 0 and girls as 1.

### 2.5. Study Attrition

We statistically investigated whether there were systematic differences between participants at all measurement points and those who responded less frequently. The tests showed that participants who responded at all measurement points were more often girls, enrolled in the general study track, and had higher socioeconomic status than the rest of the students. We also investigated whether there were significant differences in the outcome measures based on participation in all versus fewer measurement points. Pupils who responded at all measurement points experienced higher levels of class belonging and life satisfaction, less loneliness, higher grades, and less absenteeism, compared with those who responded less frequently. In contrast, we found that students who did not respond at one measurement point had more perceived teacher support than those who always responded. There was no difference between the pupils in terms of perceptions of a caring climate.

### 2.6. Main Analyses

The main analyses were performed using Stata 14 with maximum likelihood estimation. The data were normally distributed, and the latent scales were invariant over time. In order to examine the effects of the interventions, we assessed a multi-level linear mixed model that produces fixed and random effect results. The fixed effect was the impact of the interventions, while the random effect captured the residual variance. The fixed effect includes interactions between time and intervention conditions, sex, and socioeconomic status. The level of statistical significance was 0.05. It is important to note that in Norway, the school year starts in August and ends in June; therefore, life satisfaction, loneliness, and psychological distress were measured on the first measurement occasion, which took place in the beginning of year 1 (baseline), when the students had just started their upper secondary education. In contrast, teacher support, classmate support, and caring climate were first measured in the spring of year 1 (Y1), when the students had assumably gotten to know their environment, and the people within it, well enough to assess the perceived support they experienced from it.

## 3. Results

### 3.1. Teacher Support

As shown in Table 1, there was no significant effect of the interventions on perceived teacher support across time compared with the control group. Table 2 shows the students’ change in teacher support from March/April of year 1 to March/April of year 3 for I1, I2, and the control group. These results indicate that students in all groups experienced high levels of teacher support in year 1. From years 1 to 3, there were significant negative changes in perceived teacher support for students in the I1 and I2 groups.

### 3.2. Classmate Support

The results shown in Table 1 indicate no significant effects of the interventions on classmate support over time compared with the control group. The results presented in Table 2 show that students reported high levels of classmate support in year 1 and experienced minor changes from years 1 to 3, with a weak and significant reduction in I1.

### 3.3. Caring Climate

There were no significant effects of the interventions on caring climate across time compared with the control group (see Table 1). Table 2 shows that the students’ perceptions of a caring climate in their school remained high and stable throughout upper secondary school.

### 3.4. Psychological Distress

As shown in Table 1, there were no significant effects of the interventions on anxiety and depression over time compared with the control group. Table 3 shows that psychological distress significantly increased from the autumn of year 1 to the spring of year 3 in I1, I2, and the control group.

### 3.5. Life Satisfaction

Table 1 indicates no significant effects of the interventions on life satisfaction across time compared with the control group. As shown in Table 3, there was a significant decline in life satisfaction in the control group.

### 3.6. Loneliness

As shown in Table 1, there were no significant effects of the interventions on loneliness over time compared with the control group. Table 3 indicates no significant changes in loneliness in any of the groups.

### 3.7. Grade Point Average

As shown in Table 1, there were no significant effects of the interventions on the students’ grade point averages across time compared with the control group. All groups experienced an increase in their grade point average from the spring of year 1 to the spring of year 3.

### 3.8. Absenteeism

Table 1 shows no significant effects of the interventions on absenteeism over time compared with the control group. Table 4 indicates significant increases in the hours absent for all groups; however, there was only a significant increase in the days absent from the spring of year 1 to the spring of year 3 in I1.

### 3.9. Completion

Our mapping showed that 7.9% of the students in the control group (n = 522) were registered as dropouts. The dropout prevalence in the five schools ranged from 0% to 13.1%. In the I1 intervention group (n = 708), 3.2% of the students dropped out of school. The dropout rate among the I1 schools ranged from 0% to 8%. Lastly, in the I2 intervention group (n = 892), 5.4% of the students dropped out, with the rate ranging from 1.9% to 9.4% between the schools. We performed a logistic regression, wherein school ID was used as a cluster variable, to determine whether there was a significant difference between these intervention groups. The results showed no significant intervention effects. As in the effect analyses presented above, the lack of effects may be due to the low number of schools. The number of students who completed their education or started professional training was generally similar in all three groups and varied between 76.6% and 77.8%. A slightly larger number of students were enrolled in education in the intervention groups than the control group, 18–18.9% and 14.8%, respectively.

### 3.10. Dropout

During the three-year duration of this study, only 112 students from the baseline measurement dropped out. This corresponded to 5.1% of the total number of students (N = 2203). Of the 112 dropouts, 69 were registered in some form of activity (e.g., in work, work apprenticeship, follow-up services, or maternity leave), while 26 were registered as inactive (e.g., in treatment, no structured activity, or declined follow-up), and the activity of the remaining 17 was unknown. Although the low prevalence of dropout was a good thing, it prevented us from comparing the three groups in terms of their dropout rates. Most dropout students in all groups were registered as active.

## 4. Discussion

The aim of this study was to examine whether the DSP and MHST interventions impacted the students’ perceived classmate support, caring climate, teacher support, psychological distress, life satisfaction, loneliness, grade point average, absenteeism, and school completion in upper secondary school after one, two, and three years of intervention. The results showed no significant effects of either the DSP (I1) or the DSP and MHST in combination (I2) on the outcome variables.

### 4.1. The Psychosocial Learning Environment

We found no significant effects of the interventions on teacher support. Based on findings from other studies on teacher support, mental health, and drop out [9], we postulated the hypothesis that support should increase, as the measures in the intervention aim to build supportive relations among students and among students and their teachers. Interestingly, however, a small decrease in perceived teacher autonomy support in the single- and multi-tier groups was observed, which may be related to a kind of Hawthorne effect whereby an intervention explicitly focusing on relationships in the class and the school could have made students more attuned to higher expectations of teacher support than the control group.

In addition, the Norwegian school system, with many subjects taught by several teachers in different student group setups, could present a structural barrier to establishing good teacher–student relationships. In other words, it is possible that because students spend little time with each individual teacher, it is difficult to form meaningful connections [49]. However, research on instructor support in higher education, where students spend even less time with individual instructors than in secondary school, has indicated that autonomy-supportive teachers benefit students’ perceived competence, motivation, and academic functioning [50,51]. Further, previous research on upper secondary school samples has shown that relationships with teachers are important for students’ mental health [9] and completion of their education [1]. The students in our sample reported relatively high perceived teacher support in all three years of upper secondary school, indicating that students were generally content with their teacher–student relationships. This also provides the possibilities for a ceiling effect.

### 4.2. Mental Health

Regarding the results on mental health indicators (i.e., psychological distress, life satisfaction, and loneliness), we found no significant effects over the full three-year period. However, the students in all three groups consistently reported an increasing level of psychological distress, while only the control group reported a significant decrease in life satisfaction during upper secondary school. Previous research on life satisfaction has found drastic decreases during the adolescent age [52], suggesting that the lack of change in the intervention groups may be a sign of successful deceleration. However, the differences over time between these groups were small and should be interpreted with caution. An increase in mental health problems with increasing age has also been found in other surveys among upper secondary school students [53], and it has been found that for girls the prevalence of high anxiety and depressive symptoms was 30% for first- and second-year students, and 33% for third-grade students, while boys reported 11%, 12%, and 14% in first, second, and third grades, respectively. These findings underscore the importance of measures aimed at building a positive psychosocial environment and reducing mental health distress in upper secondary education. Examining the effect after the first year of the intervention, we found a significant three-way interaction between gender, measures, and time, which showed that girls in schools with the multi-tier intervention (DSP and MHST (I2)) had a lower increase in psychological distress compared with girls in the control schools and in schools in the single-tier group with only the DSP intervention (I1) [23]. According to Eccles and Roeser [25], the transition between school levels, like in this study, consists of a risk factor for social integration, and that it is important to try to ease the transition by focusing on building a good school environment. Based on the results from the present follow-up study, it seems likely that the previously found effects on girls’ mental health in the first year did not last beyond grade one. Both the DSP and MHST are whole-school approaches intended to build or reinforce a positive school culture, and as such we anticipated longer-term effects of these interventions. However, a possible explanation for the lack of such effects could be that the targeted classroom environment efforts, such as dream classes 1 and 2 of the DSP, are only implemented in year 1 and not in year 2 or 3, leaving older students less directly exposed to the systematic work of the DSP.

### 4.3. Academic Adjustment

A good psychosocial environment may contribute to learning and wellbeing [38], which, in turn, can affect both absence and subject grades. Through our analyses, we found no statistically significant effects of the measures on the development in the students’ absence or grade point average. All three groups experienced improved grade point averages and increased hours of absence during upper secondary school, while the single-tier intervention group experienced an increased number of days absent. Previous research has shown that being socially integrated and experiencing school belonging has several positive implications [6,7,8,31,35,37,38]; therefore, motivating the students to stay at school is important. Moreover, in light of the effects of COVID-19 and students’ increased level of school absenteeism and refusal worldwide [54], it might be more important than ever to work systematically with building an inclusive and good psychosocial environment where students are motivated to stay. As implicated from the results in this study, there might be a need for working systematically with higher intensity at all grade levels to build a good psychosocial environment, and not merely in the first grade of upper secondary school.

We found no significant effect of the measures on the proportion who had completed school or were in training when we compared the two intervention groups with the control group. This proportion was relatively similar in all three groups and varied between 76.6% and 77.8%. In the two intervention groups, there were slightly larger proportions of those that were still in education than in the control group, 18–18.9% and 14.8%, respectively. These figures are in line with other studies, and the proportion of students who complete school and pass within five years has remained stable since 1999 at approximately 70% (between 68% and 72%) [55].

Another important purpose for tracking all the students in our sample was to gain a nuanced picture of the students’ progress through upper secondary education. Previous research has pinpointed the importance of completing upper secondary school for gaining access to higher education [1,2], reduce risk of economic hardship [3], as well as better mental health [5] and life satisfaction [4]. Overall, we found that only 112 of the pupils we followed in our sample dropped out of upper secondary school, which corresponded to 5.1% of a total of 2203 pupils. Of these, only 1.2% were registered as not being in some form of activity or follow-up at the final time point. According to Markussen [2], students who drop out of upper secondary education early will have a greater risk of also falling out of working life. Those who complete their time in education perform better, even if they do not pass, and the longer they stay in school, the better their chances of entering working life or completing education later. A limitation of this study was that while national figures and statistics are usually based on completion after five years, we only followed the students for three. Therefore, we can only present a picture of the situation after three years. If we had followed the students for five years, our picture may have looked different, as other studies have shown that the total number of graduates is higher after five years than after three [1].

### 4.4. Limitations

Measuring the effects of interventions targeting psychosocial aspects is challenging, and the precision of self-report measures is crucial. Although great care was taken to use the appropriate measures, a contributing factor to non-significant effects could be the use of measures that did not capture existing effects. Another aspect to consider is the possibility of ceiling effects. For example, all three school-specific psychosocial constructs (teacher support, classmate support, and caring climate) displayed relatively high mean scores (3.57–4.07 on a scale from one to five) across intervention groups and years. This could indicate that the potential for improvement under school-specific conditions was limited in these schools.

Another aspect may be related to the slightly low statistical power of this study and the risk of a Type III error (i.e., rejecting the hypothesis not because there is no effect but because it cannot be detected). The initial power analyses were performed at the class level, as the interventions, especially the DSP, were mainly related to class. However, as the implementation of the DSP by the program owner was intended to be a whole-school measure, randomization was performed with schools as a cluster. Such an intervention usually yields small effect sizes (Cohen’s D = 0.20), while the power in this study could only detect moderate-to-high effect sizes (Cohen’s D = 0.50), thus resulting in the possibility of undetected effects.

### 4.5. Implications

The lack of significant effects on the study outcomes in this study does not imply that systematic work with the psychosocial learning environment is futile. Rather, more developments are needed to tailor efforts to specific psychosocial and academic outcomes, as well as to be attentive to sociodemographic characteristics, such as age and gender. As indicated by the effect studies in the COMPLETE project, a small effect on mental health was identified for girls after the first year [23], but not in the present longer follow-up study. This indicated that systematic efforts may have to be continuous and of a multi-tier character throughout upper secondary school to counteract the increasing psychological distress levels observed in girls over this age period.

Further, taking a whole-school approach, like the COMPLETE project undertook, is a demanding endeavor, but building systematic systems and cultures for a positive psychosocial learning environment that are integral parts of the school will likely be more effective than interventions that only last for a short period of time. Implementation research has shown that new efforts introduced into an organization may need three to five years to be fully integrated and successfully implemented [56,57]. Research projects evaluating large-scale efforts, like in the COMPLETE project, must be designed with sufficient time for implementation. Considering the worrying increasing trends in psychological distress, loneliness, and dropout among adolescents, transdisciplinary efforts, such as those promoted in the COMPLETE project, are warranted for future educational policies and systems. As such, the COMPLETE project, with its comprehensive and cross-sectoral and transdisciplinary approach, can serve as a model and source of learning for future efforts.

## 5. Conclusions

In conclusion, contrary to our expectations, the results of this follow-up study of a single-tier (DSP) and a multi-tier (DSP + MHST) intervention on psychological and academic adjustment outcomes indicated no significant effects compared with the control group. We underscore the importance of viewing these results in the context of the abovementioned limitations. Moreover, the school is a complex system as a setting and subject of research. Future similar studies should be attentive to the potential challenges of implementing RCTs in the school setting. Furthermore, the long-term effects of school interventions on constructs, like those examined in this study, could be difficult to observe or promote owing to the complexity of the phenomena. Although a range of school factors can influence students’ psychological wellbeing and academic functioning, there is still a range of factors outside the school arena influencing psychological distress, loneliness, life satisfaction, absence, grades, and ability to stay in school that the school setting cannot directly address. Future research could consider addressing a wider array of life domains of adolescents that are relevant for school thriving to better understand how school interventions can work effectively.

## Figures and Tables

**Table 1:** Results of the effect analyses for all outcomes, tested separately and adjusted for gender and socioeconomic status.

	Fixed Effects																Random Effects				
			Time			Socioeconomic Position		Sex	Intervention		Interaction between Time and Intervention							Multi-Level Results			
Outcome		Constant	Y1	Y2	Y3	Moderate	High	Girls	I1	I2	Y1# I1	Y1# I2	Y2# I1	Y2# I2	Y3# I1	Y3# I2		School	Class	Individual	Residual
Classmate support	Coefficient	3.77 ***		−0.17 ***	−0.12	0.25 ***	0.36 ***	−0.04	−0.02	−0.05			0.06	−0.02	−0.01	0.05	Variance	0.01	0.04	0.20	0.34
N = 3682	SE	0.09		0.05	0.07	0.06	0.05	0.03	0.09	0.09			0.06	0.06	0.08	0.08	SE	0.01	0.01	0.01	0.01
Teacher support	Coefficient	3.6 ***		−0.01	0.05	0.17**	0.29 ***	−0.23 ***	−0.05	0.04			0.04	−0.03	−0.09	−0.09	Variance	0.02	0.02	0.27	0.37
N = 3664	SE	0.01		0.03	0.04	0.06	0.06	0.03	0.11	0.1			0.06	0.06	0.08	0.08	SE	0.01	0.01	0.02	0.01
Caring climate	Coefficient	3.78 ***		−0.05	0.04	0.12*	0.25 ***	−0.12 ***	−0.07	0.03			0.05	−0.03	−0.06	−0.06	Variance	0.01	0.02	0.25	0.28
N = 3555	SE	0.09		0.04	0.06	0.05	0.05	0.03	0.1	0.1			0.06	0.06	0.07	0.07	SE	0.01	0.01	0.02	0.01
Psychological distress	Coefficient	1.91 ***	0.11 ***	0.05	0.21 ***	−0.31 ***	−0.44 **	0.51	−0.01	−0.03	−0.01	−0.03	0.13	0.13	0.07	0.06	Variance	0.01	0.01	0.30	0.26
N = 5799	SE	0.07	0.03	0.04	0.05	0.04	0.04	0.03	0.07	0.07	0.05	0.04	0.05	0.05	0.07	0.06	SE	0.00	0.00	0.01	0.01
Life satisfaction	Coefficient	2.67 ***	−0.09 *	−0.08*	−0.15 ***	0.25 ***	0.45 ***	−0.22 ***	−0.01	0.02	0.02	0.02	0.03	−0.02	0.07	0.07	Variance	0.00	0.01	0.20	0.19
N = 5730	SE	0.06	0.03	0.04	0.05	0.04	0.04	0.02	0.06	0.06	0.04	0.04	0.04	0.04	0.06	0.06	SE	0.00	0.00	0.01	0.00
Loneliness	Coefficient	2.55 ***	0.08 *	0.08	0.08	−0.33 ***	−0.50 ***	0.22 ***	−0.01	0.02	0.01	−0.08	0.01	0.02	0.05	0.00	Variance	0.00	0.02	0.32	0.25
N = 5706	SE	0.07	0.03	0.04	0.05	0.04	0.04	0.03	0.07	0.07	0.05	0.04	0.05	0.05	0.06	0.06	SE	0.00	0.01	0.01	0.01
Grade point average	Coefficient	3.98 ***		−0.05	0.07	0.03	0.04	0.23 ***	0.06	−0.05			0.07	0.03	−0.02	−0.05	Variance	0.01	0.08	0.47	0.15
N = 3827	SE	0.1		0.03	0.04	0.05	0.05	0.04	0.12	0.11			0.04	0.04	0.05	0.05	SE	0.01	0.02	0.02	0.00
Hours absent	Coefficient	15.67 ***		2.23 *	4.44 **	−3.2 *	−4.95 ***	0.17	−0.36	−1.58			1.39	−1.02	1.68	0.96	Variance	0.40	17.53	131.59	193.24
N = 3770	SE	1.7		1.09	1.51	1.33	1.31	0.77	1.56	1.47			1.44	1.39	1.83	1.83	SE	2.26	4.72	9.18	6.57
Days absent	Coefficient	5.54 ***		0.09	0.1	−1.49 **	−1.91 ***	0.97 ***	0.15	0.55			−0.62	−0.05	0.48	0.80	Variance	0.57	2.52	21.04	22.87
N = 3770	SE	0.72		0.38	0.53	0.48	0.47	0.29	0.76	0.73			0.50	0.48	0.64	0.64	SE	0.43	0.70	1.38	0.82

Notes: SE = standard error, Y = year, I = intervention, and # = interaction. * *p* < 0.05, ** *p* < 0.01, and *** *p* < 0.001. Classmate support, teacher support, and caring climate were not part of Y1; this was also the case for grade point average and absents.

**Table 2:** Descriptive statistics for and changes in teacher support, classmate support, and caring climate.

Variable	Group	Spring of Y1		Spring of Y2		Spring of Y3		Spring of Y1–Spring of Y3
		n	M (SD)	n	M (SD)	n	M (SD)	Change
Teacher support	C	430	3.76 (0.81)	293	3.76 (0.93)	124	3.73 (0.89)	−0.03
	I1	558	3.69 (0.84)	432	3.74 (0.80)	290	3.57 (0.87)	−0.19 *
	I2	774	3.81 (0.77)	482	3.79 (0.85)	296	3.71 (0.79)	−0.10 *
Classmate support	C	436	4.05 (0.81)	297	3.91 (0.87)	127	4.05 (0.73)	0.00
	I1	594	4.07 (0.72)	428	3.99 (0.75)	292	3.97 (0.73)	−0.10 *
	I2	771	4.02 (0.71)	488	3.85 (0.80)	293	3.99 (0.70)	−0.03
Caring climate	C	415	3.94 (0.77)	280	3.91 (0.85)	123	3.99 (0.77)	0.02
	I1	568	3.87 (0.73)	423	3.91 (0.71)	289	3.84 (0.73)	−0.03
	I2	747	4.00 (0.73)	466	3.94 (0.76)	289	3.96 (0.71)	−0.04

Notes: Y = year, C = control, I = intervention, M = mean, and SD = standard deviation. * *p* ≤ 0.001.

**Table 3:** Descriptive statistics for and changes in anxiety and depression, life satisfaction, and loneliness.

Variable	Group	Autumn of Y1		Spring of Y1		Spring of Y2		Spring of Y3		Autumn of Y1–Spring of Y3
		n	M (SD)	n	M (SD)	n	M (SD)	n	M (SD)	Change
Psychological distress	C	490	1.84 (0.82)	441	1.93 (0.85)	308	1.83 (0.78)	138	2.09 (0.82)	0.25 **
	I1	684	1.78 (0.78)	597	1.86 (0.80)	438	1.89 (0.79)	305	2.06 (0.76)	0.28 **
	I2	887	1.74 (0.79)	768	1.80 (0.78)	493	1.87 (0.82)	310	2.09 (0.83)	0.35 ***
Life satisfaction	C	490	2.90 (0.64)	431	2.83 (0.70)	304	2.86 (0.68)	228	2.78 (0.68)	−0.12 *
	I1	676	2.94 (0.66)	587	2.91 (0.68)	432	2.95 (0.71)	406	2.89 (0.65)	−0.05
	I2	871	2.96 (0.64)	767	2.90 (0.65)	489	2.90 (0.67)	457	2.89 (0.67)	−0.07
Loneliness	C	478	2.25 (0.78)	434	2.33 (0.80)	295	2.29 (0.80)	139	2.29 (0.78)	0.04
	I1	662	2.21 (0.78)	585	2.26 (0.77)	435	2.21 (0.78)	306	2.28 (0.81)	0.07
	I2	866	2.24 (0.79)	768	2.23 (0.77)	485	2.29 (0.78)	305	2.29 (0.81)	0.05

Notes: Y = year, C = control, I = intervention, M = mean, and SD = standard deviation. * *p* < 0.05, ** *p* < 0.01, and *** *p* < 0.001.

**Table 4:** Descriptive statistics for and changes in hours and days absent and grade point average.

Variable	Group	Spring of Y1		Spring of Y2		Spring of Y3		Spring of Y1–Spring of Y3
		n	M (SD)	n	M (SD)	n	M (SD)	Change
Hours absent	C	480	15.6 (27.7)	431	14.5 (23.7)	221	20.5 (27.9)	4.9 *
	I1	677	14.8 (27.5)	521	16.8 (26.3)	449	19.5 (26.1)	5.3 ***
	I2	910	11.8 (22.7)	729	11.9 (15.2)	460	16.6 (19.4)	4.8 ***
Days absent	C	480	6.4 (12.3)	431	5.3 (9.1)	221	5.9 (9.8)	−0.3
	I1	677	4.8 (7.3)	521	4.2 (7.3)	449	6.1 (9.8)	1.2 **
	I2	910	6.5 (12.0)	729	5.9 (11.1)	460	6.0 (10.4)	0.7
Grade point average	C	511	4.01 (1.04)	407	4.13 (0.93)	206	4.31 (0.99)	0.30 ***
	I1	694	4.17 (0.85)	512	4.17 (0.86)	440	4.26 (0.94)	0.09 *
	I2	887	4.04 (0.85)	737	4.05 (0.92)	439	4.21 (1.00)	0.17 ***

Notes: Y = year, C = control, I = intervention, M = mean, and SD = standard deviation. * *p* < 0.05, ** *p* < 0.01, and *** *p* < 0.001.

## Data Availability

The data presented in this study are available on request from the corresponding author. The data are not publicly available due to ethical restrictions.
